# Supplementary material for: Whole Genome Sequencing of the Novel Probiotic Strain Lactiplantibacillus plantarum FCa3L
Source: Microorganisms. 2023 May 7;11(5):1234. doi: 10.3390/microorganisms11051234 (PMC10224263; doi:10.3390/microorganisms11051234)
Supplement: Supplementary file 1 [file microorganisms-11-01234-s001.zip › microorganisms-2381077-supplementary.pdf]

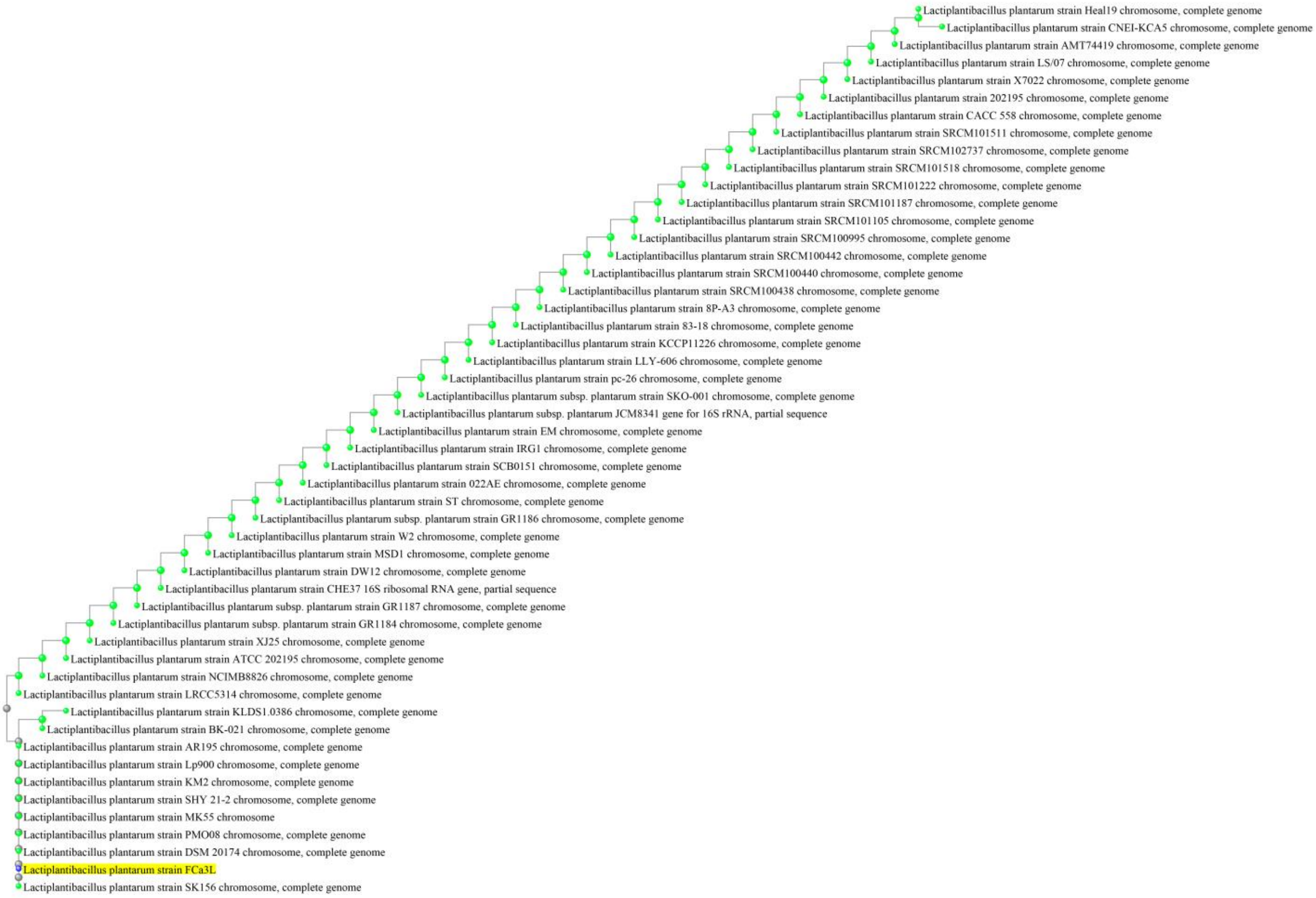

Figure S1. The whole genome-based phylogenetic tree of *L. plantarum* FCa3L generated by EMBL-EBI server (https://www.ebi.ac.uk/Tools/phylogeny/simple_phylogeny/ accessed on) using the whole-genome sequences of the *L. plantarum* species.
